# The Influence of the Relative Timing between Pole and Heel Strike on Lower Limb Loading among Young and Older Naïve Pole Walkers

**DOI:** 10.1155/2022/3938075

**Published:** 2022-07-07

**Authors:** Vincenzo E. Di Bacco, Jeevaka B. Kiriella, William H. Gage

**Affiliations:** School of Kinesiology and Health Science, York University, Toronto, ON, Canada

## Abstract

Current research is unclear with respect to whether pole walking (PW) reduces lower limb loading when compared to regular walking (RW). Contradictory findings in the literature may be related to the relative timing between pole and foot contact events, which were examined in the current study among naïve pole walkers. Fourteen young (4 F; 25.3 ± 5.4 years) and 8 older adults (4 F; 68.5 ± 3.2 years) performed PW and RW trials along a force plate embedded walkway at two different visits. The time difference between pole and foot contact during both the onset of ground contact and the peak force application was calculated. Several kinetic measures were calculated for the lower limbs and poles. A significant decrease during PW, compared to RW, was found for foot impulse (2.1%; *p* < 0.01), peak vertical ground reaction force (vGRF) (3.4%; *p* < 0.01), rate of loading (5.2%; *p*=0.02), and peak push-off vGRF (2.1%; *p*=0.01). No difference in pole loading was found between age groups and visits. No significant correlations were found between the relative timing and foot loading measures. Significant low-to-moderate negative correlations were found between peak foot and pole vGRFs (*p*=0.04), peak foot vGRF and pole strike impulse (*p*=0.01), peak foot vGRF and pole impulse (*p*=0.02), and peak foot push-off vGRF and pole impulse (*p*=0.01), suggesting that as pole loading increased, foot loading decreased. Findings suggest timing between pole and heel contact may not be related to unloading the lower limbs but may be related to other aspects of pole use since PW reduced lower limb loading.

## 1. Introduction

Pole walking (PW) has become popular as a form of physical activity in many communities worldwide. Researchers have described and reported numerous health benefits of PW, including reduced blood pressure, increased activity levels, and improved self-reported quality of life [1]. PW has been demonstrated to provide additional health benefits when compared with regular walking (RW) associated primarily with the use of the upper body and upper limbs during walking, using specialized poles, whereby the arms are used to plant poles simultaneously with each step, in a manner similar to cross-country skiing [2]. The utility of learning and practicing PW for bringing about additional health and mobility benefits has also been widely demonstrated in various clinical populations such as in older adults and patients with Parkinson's disease and intermittent claudication [3–11].

Previous research has advocated PW as a potential rehabilitation intervention for offloading lower limb joints and allowing patients to continue active lifestyles [12]. Reduced loading at the lower limbs may prevent joint degeneration and development or progression of osteoarthritis [13]. However, investigations of forces and loading during PW have provided conflicting results, including reduced joint loads [14–16], no changes in joint loads [17, 18], and increased joint loading [19], compared to RW. Willson et al. [14] investigated a group of young novice participants and found an average decrease in vertical GRF and vertical knee joint reaction force during PW compared to RW. Hansen et al. [18] reported that PW and RW did not differ in terms of peak knee joint compression and shear forces, as well as GRFs among experienced middle-aged pole walkers. Encarnación-Martínez et al. [20] investigated a group of young experienced pole walkers and found that the GRFs during PW were 27% higher at the instant of heel strike and 8% lower at takeoff, compared to RW. Similar results during heel strike were found by Brunelle and Miller [21], who reported an increase of ∼26% for vertical GRF and ∼6% for anterior-posterior GRF during PW. Among young experienced pole walkers, Steif et al. [19] found larger knee joint loads in all directions at heel contact during PW and also reported that the vertical ground reaction force (vGRF) did not change with the use of poles.

During PW, the load applied to the poles is between 30 and 50 N [22–24]. Jensen et al. [25] manipulated pole loading to investigate the effect on knee joint compression force. The peak pole force during normal walking was ∼7% of body weight (BW), and during increased pole force, it was ∼17% of BW. Although pole force was 2.4 times greater in the increased-load condition, there was no change in resultant GRFs at heel contact or knee joint compressive force. These findings suggest that participants using walking poles were unable to apply large enough forces to support the body weight and reduce loading of the lower limbs, or that the positioning and orientation of the walking poles, not typically being orthogonal to the ground, reduced the normal force at the ground. However, no research has examined the relative timing between foot and pole contact. The relative timing of foot and pole ground contact could influence any contribution made by the pole to reducing lower limb joint loading. The objectives of this study were to (1) explore the relative timing of pole and foot GRFs among young and older naïve pole walkers, (2) compare pole loading forces among young and older naïve pole walkers, (3) compare the effects of PW on foot loading forces among young and older naïve pole walkers, and (4) determine whether there is a significant correlation between the relative time difference and foot loading forces and a significant correlation between pole and foot loading forces.

## 2. Materials and Methods

### 2.1. Participants

Twenty-two people volunteered to participate in the study, including 14 healthy young adults (YA) (4 females, 10 males; mean ± SD; age: 25.3 ± 5.4 years, body mass: 74 ± 14 kg, body height: 1.76 ± 0.09 m) and 8 healthy older adults (OA) (4 males, 4 females; mean; age: 68.5 ± 3.2 years, body mass: 74.7 ± 10.1 kg, body height: 1.66 ± 0.07 m). Each participant provided informed consent prior to participation. The institutional research ethics review board granted approval (REB #e2014-292) for the study. Exclusion criteria included previous pole walking experience and any previous neurological or orthopedic conditions within the past six months that might have affected gait performance.

### 2.2. Equipment/Instrumentation

Four force plates (AMTI, Watertown, MA, USA) were embedded in a 7-meter long walkway. The force plates were positioned in sets of two and were used to collect ground reaction forces. Two walking poles (Nordixx, TO, CA) were used. All force plate data were recorded using the Vicon Nexus (Version 1.6.1) software and stored on a dedicated laptop for later processing.

### 2.3. Protocol

Participants visited the laboratory twice, with seven days between visits. Participants wore a black tank top, shorts, and shoes throughout testing. During the first visit (V1), all participants were provided with a standard set of instructions on pole walking; pole length was adjusted based on each participant's height. The instructions included the condition of timing heel contact to pole contact with the ground. Participants watched a video, provided by the walking pole manufacturer (Nordixx, TO, CA), for the purpose of providing initial instructions and a demonstration of pole walking technique. Participants also completed a series of practice trials, stopping when they self-reported familiarity with and comfort using the walking poles. Participants were asked to perform six randomly ordered blocks of five walking trials, either with or without poles, for a total of 30 trials (15 trials with and 15 without poles), while walking at a self-selected pace. Participants stepped on the force plates in series, such that a single footfall and single pole strike occurred on each force plate. Participants completed approximately four steps prior to the force plates and four steps following the force plates, while walking along the 7-meter-long walkway. The same testing protocol was completed by each participant on the second visit (V2); however, participants did not watch the video and did not perform practice trials during the second visit. The same experimental set-up and equipment, including the same walking poles, force plates, and walkway surfaces, were used with all participants on both visits.

### 2.4. Data Processing and Dependent Measures

All force plate data, originally sampled at 1000 Hz, were digitally filtered (Visual 3D v. 5, C-Motion Inc., ON, Canada) using a fourth order low-pass Butterworth filter, with a cutoff frequency of 6 Hz. The filtered force plate signals were used to identify the onset of heel and pole strike events during walking trials, which were selected at the instant of an increase in vertical ground reaction force (vGRF) greater than 10 Newtons. Step time was calculated as the time difference (seconds) between the onset of the vGRF of the first step on the force plate and the onset of the vGRF of the second step on the following force plate. Although two steps were captured on the force plates, the data for step one were presented.

The relative time between heel strike and pole strike was determined as the time difference between heel strike onset and pole strike onset events (ms). Delta1_onset_ (D1_onset_) represented the time difference between the first step and the first pole strike. A value of 0 ms would indicate that heel strike and pole strike occurred simultaneously during PW. A positive value indicated heel strike occurred prior to pole strike.

The force plate signals were used to identify the peak amplitude of the vGRF of the first step (F1_peak-vGRF_) and the peak amplitude of the vGRF under the walking pole (P1_peak-vGRF_).

The relative time between peak heel strike and peak pole strike was determined as the time difference between the peak amplitude of the vGRF of heel strike and the peak amplitude of the vGRF of pole strike events (ms). Delta1_peak_ (D1_peak_) represented the time difference between the first step and the first pole strike. A value of 0 ms would indicate that peak heel strike and peak pole strike occurred simultaneously during PW. A positive value indicated peak heel strike occurred prior to peak pole strike.

The impulse (N∗s) of heel strike one (HS1_impulse_) and pole strike one (PS1_impulse_) was calculated as the integral between vGRF onset and peak vGRF events during step one. The impulse of the entirety of step one footfall (F1_impulse_) and pole contact (P1_impulse_) was calculated as the integral between vGRF onset and vGRF toe-off or pole-off events. Toe-off and pole-off events were identified as the instant where the vGRF force plate signal decreased below 10 N. The push-off force for step one (F1_push-off_) was identified as the peak vGRF between peak heel strike and toe-off events.

The rate of loading (ROL) (N/s) of heel strike one (HS1_ROL_) and pole strike one (PS1_ROL_) was calculated as the change in vGRF divided by the change in time between onset and peak vGRF events during step one. All vGRF foot measures derived from step one were normalized to 100% body weight for each participant.

### 2.5. Data Removal and Categorization

From the 1320 trials collected across all participants and both visits, 660 trials were PW and 660 trials were RW. The vGRF profiles of each footfall and pole strike during step one on the force plates for each trial were visually inspected for erroneous trials, which included stepping on both force plates with the same foot, not placing each footfall or pole strike entirely on the force plate, or not achieving the onset threshold of 10 N. From the 660 RW trials, 641 trials (YA = 406; OA = 235) for step 1 were included for analysis. From the 660 PW trials, 584 trials (YA = 379; OA = 205) for step 1 were included for analysis.

**Figure 1:**
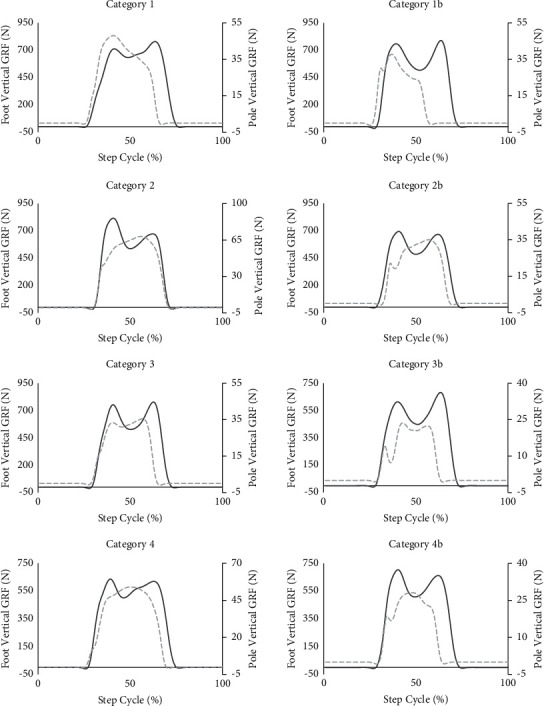
Representative plots of all pole force profile categories. The plots represent a single step during pole walking over the force plates. The dark grey solid line represents the vertical GRF (vGRF) foot profile, and the light grey dashed line represents the vGRF pole profile. Both foot and pole force signals were normalized to 100% of the step cycle, defined as the period from 0.5 seconds before heel strike (0%) to 0.5 s after foot push-off (100%). Category 1 was defined as peak pole force occurring near peak heel strike force; category 2 was defined as peak pole force occurring near peak foot push-off force; category 3 was defined as a distinctly bimodal pattern in the pole GRF with forces with the two peaks occurring near the peaks of the bimodal vertical GRF associated with weight acceptance and foot push-off force; category 4 was defined as peak pole force occurring between peak heel strike and peak foot push-off force; categories 1b–4 b have the same pole force profile as their corresponding number listed above except for a transient peak present prior to peak pole vGRF. With visual inspection, it became clear that pole strike sometimes preceded heel strike and foot push-off sometimes preceded pole-off; therefore, the selection of 0.5 seconds before and 0.5 seconds after heel strike and foot push was used to ensure the entirety of the associated vertical GRF profiles was examined.

Upon visual inspection of PW trials, it became apparent that distinctly different pole force patterns emerged, even though all participants received the same instructions during V1. Therefore, PW trials were categorized based on the profile of the vGRF produced by the pole. Four categories (1, 2, 3, 4) were created based on the location of peak vGRF of the pole in relation to the footfall vGRF profile of the same step. An additional four categories (1 b, 2 b, 3 b, 4 b) were created based on the same criterion, with the addition that the vGRF of the pole also demonstrated a transient peak evident before peak vGRF (Figure 1).

### 2.6. Statistical Analysis

Statistical analyses were performed using JMP v. 9.0 software (the SAS Institute, Cary, NC, USA).

  Section 1: this section includes frequency table based on pole category (Table 1).
  Section 2: this section includes RW trials and PW trials only from categories 1, 1 b, 3, and 3 b due to the location of peak vertical pole force relative to peak vertical foot force, when foot loading forces are typically greatest (i.e., heel contact event) and therefore may assist in reducing foot loading. Additionally, these categories were included as they reflected the instructions to time heel contact to pole contact. One analysis of variance (ANOVA; 2 × 2 × 2 mixed model) was used to determine the effects of group (YA/OA), visit (V1/V2), and condition (PW/RW) on step time. Analyses of covariance (ANCOVAs; 2 × 2 mixed model) were used to determine the effects of group (YA/OA) and visit (V1/V2) for each relative time difference measure. ANCOVAs (2 × 2 mixed model) were used to determine the effects of group (YA/OA) and visit (V1/V2) for each pole force measure. ANCOVAs (2 × 2 × 2 mixed model) were used to determine the effects of group (YA/OA), visit (V1/V2), and condition (PW/RW) for each foot force measure. Step time was used as the covariate in all ANCOVA models. Normality of data was assessed visually by plotting the distribution of each measure and numerically using the Shapiro–Wilk test. Non-normal data was square-root-transformed prior to statistical analyses. Results were statistically significant at *p* < 0.05. LSMeans differences Student's *t*-test was used to compare means and test interactions when necessary.
  Section 3: partial Spearman correlation (*ρ*_partial_) coefficients, calculated using participant means, were used to determine the strength and direction of correlations, while controlling for walking speed with step time as a covariate, between the relative time difference and foot loading metrics, as well as between pole loading and foot loading metrics. The nonparametric partial Spearman correlation tests were performed due to a lack of normality and linearity, which was assessed visually. The size of the correlation coefficient was interpreted as follows: negligible = 0 to 0.3; low = 0.3 to 0.5; moderate = 0.5 to 0.7; high = 0.7 to 0.9; very high = 0.9 to 1 [26]. Results were statistically significant at *p* < 0.05.

## 3. Results

Section 1: frequency table of pole category.

Section 2: step time anova. No significant interactions were found for group, visit, and condition effects or for the main effect of group for step time. A significant main effect of visit (*F* (1, 20) = 6.49, *p* < 0.02) and a significant main effect of condition (*F* (1, 20) = 37.18, *p* < 0.01) were found for step time. All main and interaction effects are described in Table 2. All descriptive statistics are presented in Table 3.

### 3.1. Foot Force ANCOVAs

**Figure 2:**
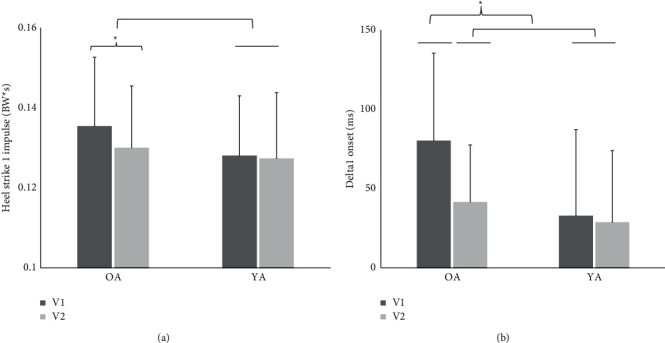
The LSMeans ± SD of (a) heel strike 1 impulse and (b) Delta1 onset of each adult group separated by visits. Data are averaged across repeated trials and participants. The dark-shaded bar represents LSMeans during visit 1 and the light-shaded bar represents LSMeans during visit 2. OA and YA represent older adult and younger adult groups, respectively. Visit 1 was significantly different from visit 2 for the OA group, which was not different from the YA group for heel strike 1 impulse. Visit 1 for the OA group was significantly different from visit 2 and YA group, which were not different from each other for Delta1 onset. Error bars represent standard deviations. Asterisk represents significance at 0.05 level.

A significant interaction effect of group and visit (*F* (1, 20) = 7.61, *p*=0.01) was found for HS1_impulse_. HS1_impulse_ was significantly lower during V2 than during V1, for older adults; younger adults did not differ between V1 and V2 (Figure 2). No other significant interactions were found for any combination of group, visit, and condition effects (Table 2). All main and interaction effects are described in Table 2. All descriptive statistics are provided in Table 3.

### 3.2. Pole Forces and Relative Time Difference ANCOVAs

A significant interaction effect of group and visit (*F* (1, 20) = 12.46, *p* < 0.01) was found for D1_onset_. D1_onset_ was significantly lower during V2, compared to V1, for older adults; younger adults did not differ between V1 and V2 (Figure 2(b)). No other significant interactions were found for group and visit effects (Table 4). All main and interaction effects are described in Table 4. All descriptive statistics are given in Table 5.

Section 3: partial correlations. Significant negative partial correlations were found between F1_peak-vGRF_ and P1_peak-vGRF_ (*ρ*_partial_ = −0.44, *p* value = 0.04), between F1_peak-vGRF_ and PS1_impulse_ (*ρ*_partial_ = −0.57, *p* value = 0.01), between F1_push-off_ and PS1_impulse_ (*ρ*_partial_ = −0.49, *p* value = 0.02), between F1_peak-vGRF_ and P1_impulse_ (*ρ*_partial_ = −0.51, *p* value = 0.02), and between F1_push-off_ and P1_impulse_ (*ρ*_partial_ = −0.58, *p* value = 0.01). All other correlations were not significant (Table 6).

## 4. Discussion

Previous research on the utility of walking poles for unloading the lower limbs has demonstrated contradictory findings, suggesting either an increase [19], a decrease [14, 16], or no change [17, 18] when using poles. While it is possible that the timing of pole contact relative to heel strike could influence the impact that loading through the walking pole might have on lower limb loading, no research to date has reported such measures. The current study investigated the time difference between pole and foot contact, among young and older naïve pole walkers, during both the onset of ground contact and the peak force application. The results revealed no significant correlations between the foot loading forces and the relative timing of pole and foot contact events, even after visually examining and excluding trials during which peak pole and foot vertical ground reaction forces were not coincident, suggesting the timing between pole and foot contact may not be related to the unloading of the lower limbs, among naïve pole walkers. However, significant low-to-moderate negative correlations were found between several pole and foot loading force measures, suggesting that pole use, in some capacity, may be related to reducing lower limb loading. Additionally, several foot loading measures demonstrated significant differences between gait conditions with walking typically demonstrating greater magnitudes of loading, when compared to pole walking. The absence of differences found for pole loading force measures between adult populations investigated or between visits suggests all participants similarly applied forces to the ground. Interestingly, distinct vertical pole loading force profiles emerged (Figure 1), even though all participants received the same instructions.

### 4.1. Relative Timing

A significant interaction effect of group and visit was found for Delta1_onset_, revealing a decrease in Delta1_onset_ between visits for only the older adult group (Figure 2(a)). A similar pattern was found for heel strike impulse, in which a decrease between visits was found for only the older adult group, irrespective of gait condition (Figure 2(b)). Together, these results demonstrate that the older adult group started at a larger value for both Delta1_onset_ and heel strike impulse and then decreased the value of these measures between V1 and V2. The decrease in Delta1_onset_ values for older adults may suggest an improvement in pole walking skill or comfort between visits (V1 average: 80 ms; V2 average: 41 ms), indicating the onset of foot contact occurred prior to the onset of pole contact. Delta1_peak_ demonstrated a significant increase for both groups between visits (V1 average: −2.5 ms; V2 average: −31.2 ms) and indicated peak pole strike force was achieved prior to peak heel strike force. No significant correlations were found between relative timing and vGRFs at the foot during pole walking, suggesting the relative time metric is not related to unloading vGRFs at the foot. Previous research has demonstrated that a minimum of eight weeks of training is required to learn pole walking technique among older adults [27] Furthermore, Pellegrini et al. [28] investigated slight deviations in Nordic walking technique compared to what is considered correct Nordic walking technique and reported a decrease in duration of pole contact time and pole force effectiveness when incorrect Nordic walking technique was used. Although learning pole walking technique was not an objective of this study, it is reasonable to suggest that pole walking experience as well as pole walking technique influences the relative timing metrics and may help explain the contradictory findings found in the literature in terms of unloading the lower limbs.

### 4.2. Pole Loading Forces

The current study revealed a peak pole strike vGRF of ∼33.5 N, collapsed across group and visit, which is consistent with the values reported in previous studies [22–24]. The magnitude of pole force application with the ground is indeed marginal when compared to the entire body weight of an individual in order to unload the lower limbs as pole force is being applied with the hands while the arms are extended with each step. The current study revealed an average pole impulse of 11.6 N∗s, similar to the impulse of recreational PWs (11.2 N∗s) previously reported by Fujita et al. [24]. An average pole strike rate of loading of 190.4 N/s was found in the current study. Schiffer et al. [23] reported a pole strike rate of loading of 487 N/s as pole walking instructors walked along a concrete surface. Since the rate of loading estimates the effort of pole plant, due to the experience level of pole walkers that participated in Schiffer et al. study [23], perhaps the pole walking instructors were able to better utilize the walking poles by applying greater effort to the poles compared to the naïve walkers used in the current study.

### 4.3. Foot Loading Forces

The majority of foot loading measures revealed significant differences between walking conditions with pole walking demonstrating lower foot loading forces than walking. This finding suggests that pole walking may aid in unloading the lower limbs, even among naïve pole walkers, and is in line with previous research [14]. For example, Hagen et al. [29] reported a 3% reduction in peak vGRF of foot push-off during pole walking when compared to walking while a 2.1% reduction was found in the current study. Encarnación-Martínez et al. [20] reported an 8.2% reduction in peak vGRF of foot push-off during pole walking compared to walking. However, the reduction in peak vGRF of heel strike and heel strike rate of loading found in the current study is contrary to previous research. Peak vGRF of heel strike and heel strike rate of loading are typically found to be significantly greater during pole walking when compared to walking [19, 20]. For example, Steif et al. [19] demonstrated a 9.2% increase in heel strike rate of loading during pole walking compared to walking, while the current study revealed a 5.2% difference with walking found to be greater than pole walking. However, the majority of pole walking studies are conducted with experienced pole walkers as the participants [19]. Additionally, previous research reports that walking poles enable participants to walk faster [14], and therefore it may become difficult to control and adequately compare pole walking and walking. For example, Encarnación-Martínez et al. [20] removed walking trials that were ±5% beyond participant's preferred speed. In the current study, participants were completely naïve to pole walking technique, and gait speed was controlled with the step time covariate. Therefore, the differences in methodology found between the previous research and the current study may explain the reduced foot loading forces found in the current study.

Although the relative time metrics in the current study did not correlate with foot loading forces, the correlation analysis revealed significant negative low-to-moderate partial correlations between peak vGRF of heel strike and peak vGRF of pole strike (−0.44), peak vGRF of heel strike and pole strike impulse (−0.57), and peak vGRF of heel strike and pole impulse (−0.51), as well as between peak vGRF of foot push-off and pole strike impulse (−0.49) and peak vGRF of foot push-off and pole impulse (−0.58), suggesting that as pole force application or duration of pole contact increases, foot loading may decrease, potentially explaining the smaller forces during pole walking compared to walking as observed in peak vGRF of heel strike and peak vGRF of foot push-off measures. A similar pattern of relationships, exhibited in the 4^th^ and 6^th^ correlation blocks of Table 6, was found between pole impulse measures (pole strike impulse and pole impulse) and peak foot force measures (peak vGRF of heel strike and peak vGRF of foot push-off). This pattern suggests that something else may be occurring between the beginning and end of the stance phase, while participants are loading and unloading with the poles. The lack of correlation between foot impulse and pole loading measures suggests that although foot impulse revealed a significant difference between gait conditions (pole walking < walking), the reduced foot loading found during pole walking may also be influenced by another element besides pole force application. Willson et al. [14] found a significant decrease in the average vGRF between pole walking and walking across the stance phase of the gait cycle. In the current study, foot impulse, which is calculated as a function of force and time throughout the stance phase, also demonstrated a significant decrease of ∼2.1% during pole walking. Although pole strike impulse and pole impulse demonstrated significant negative correlations with several foot loading measures, pole use was not related to foot impulse in pole force application. Therefore, perhaps the reduction in foot loading forces may have more to do with differences in the intersegmental dynamics between pole walking and walking.

The kinetics of the lower limbs during steady-state gait is strongly influenced by arm swing [30]. For example, Yang et al. [31] found that constrained arm swing during steady-state gait significantly increased vGRFs, when compared to unconstrained arm swing among young adults. De Graaf et al. [32] found that slightly increasing arm swing amplitude was shown to decrease ground reaction moment during walking. Furthermore, Gomenuka et al. [10] reported greater internal mechanical work related to a larger range of arm movement during pole walking compared to free walking, following an eight-week training intervention among older adults, suggesting pole walking may increase arm swing movement, compared to regular walking. Although arm swing amplitude was not measured in the current study, perhaps pole walking produced a greater arm swing amplitude when compared to walking, leading to the decreased foot loading forces found in the current study. Pellegrini et al. [33] investigated the effect of walking poles on the potential and kinetic energy fluctuations related to the center of mass displacement and found a 10.9% larger pendular recovery between these energies, likely due to pole swing and pole propulsion. Additionally, less mechanical work for moving the legs was found during pole walking, compared to walking. These findings are similar to those reported by Leal-Nascimento et al. [34], demonstrating a decrease in external and vertical mechanical work during pole walking, compared to free walking, among healthy older adults walking at 4.7 km/h. Although the total mechanical work was found to be greater during pole walking, compared to free walking among older adults, total work was greater due to the use of poles which increased internal work from the arms. Furthermore, increasing or decreasing pole length by ±5 cm [35] or even increasing pole force application to the ground by 2.4 times normal pole force [23] revealed no changes in lower limb forces during pole walking. Together, these findings suggest the use of walking poles may have more to do with altering the intersegmental dynamics, specifically arm swing, when compared to walking, as opposed to force application, and may help elucidate the reduction in foot loading forces as evidenced in the current study.

### 4.4. Perspective

Proponents of PW suggest that walking poles facilitate a more effective form of physical activity, while also being less demanding, in terms of loading, on the lower limbs, compared to RW [12]. The current study revealed that the use of walking poles reduced vGRFs at the foot among naïve pole walkers. Several pole and foot loading forces demonstrated significant low-to-moderate negative correlations, suggesting that as pole force magnitude or duration of force application increased, foot loading forces may have decreased. Additionally, it is suggested that intersegmental dynamics, perhaps related to arm swing, may play a role in unloading the lower limbs during PW as foot loading forces decreased throughout the stance phase. These findings add to the knowledge base regarding the use of walking poles and support previous claims that the use of walking poles can reduce lower limb loading [14, 16, 35]. However, more work needs to be done on the mechanism as to how walking poles reduce loading on the lower limbs. Although the relative timing between pole and foot contact revealed no relationship with any foot loading forces, future research should investigate the relative timing among experienced pole walkers and its effect on lower limb loading.

## Figures and Tables

**Table 1:** Number of pole walking (PW) trials and percent (%) of total PW trials in the older adult (OA) and young adult (YA) groups, by step category. Note. The bold values represent the categories that were included in Sections 2 and 3 of the statistical analysis, based on the location of peak vertical pole force relative to peak vertical foot force, when foot loading forces are typically the greatest.

Step 1 category	Group	
	OA	YA
	N (% of total PW trials)	N (% of total PW trials)
1	**119 (58.0)**	**163 (43.0)**
1b	**13 (6.3)**	**98 (25.9)**
2	1 (0.5)	5 (1.3)
2b	0 (0.0)	16 (4.2)
3	**14 (6.8)**	**42 (11.1)**
3b	**7 (3.4)**	**24 (6.3)**
4	46 (22.4)	15 (4.0)
4b	5 (2.4)	16 (4.2)
Total	205	379

**Table 2:** ANOVA results of step time and ANCOVA results of step 1 foot force measures with step time as the covariate.

Measure	Group	Visit	Condition	Group × visit	Group × condition	Visit × condition	Group × visit × condition
Step time (s)	0.29	**0.02** ^*∗*^	<0.01^*∗*^	0.97	0.08	0.45	0.96
Foot 1 impulse (BW∗s)	0.48	0.78	**<0.01** ^*∗*^	0.42	0.26	0.58	0.23
Foot 1 peak VGRF (BW)	**0.04** ^*∗*^	0.75	**<0.01** ^*∗*^	0.90	0.54	0.66	0.59
Heel strike 1 impulse (BW∗s)	0.28	**0.01** ^*∗*^	0.84	0.01^*∗*^	0.08	0.48	0.40
Heel strike 1 ROL (BW/s)	**0.01** ^*∗*^	0.34	**0.02** ^*∗*^	0.64	0.58	0.20	0.95
Foot 1 push-off peak VGRF (BW)	**0.02** ^*∗*^	0.03^*∗*^	**0.01** ^*∗*^	0.74	0.19	0.06	0.90

*Note.* ANOVA: analysis of variance, ANCOVA: analysis of covariance. Bold *p* values represent larger mean values for visit 1 compared to visit 2; young adult compared to older adult; walking compared to pole walking for the corresponding measure. ^*∗*^Statistical significance at *p* < 0.05.

**Table 3:** Summary of descriptive statistics (LSMeans ± SD) for step time and all foot force measures separated by group, visit, and condition.

Group	Visit	Condition	Step time (s)	F1 impulse (BW∗s)	F1 peak vGRF (BW)	HS1 impulse (BW∗s)	HS1 ROL (BW/s)	F1 push-off (BW)
OA	V1	PW	0.642 ± 0.0629	0.576 ± 0.071	1.029 ± 0.077	0.137 ± 0.0173	4.823 ± 0.963	1.001 ± 0.045
		RW	0.581 ± 0.0515	0.584 ± 0.055	1.057 ± 0.063	0.134 ± 0.0143	4.974 ± 0.879	1.041 ± 0.055
	V2	PW	0.623 ± 0.0539	0.576 ± 0.054	1.027 ± 0.084	0.131 ± 0.0150	4.916 ± 1.006	1.017 ± 0.058
		RW	0.569 ± 0.0531	0.587 ± 0.054	1.063 ± 0.065	0.129 ± 0.0131	5.222 ± 0.903	1.044 ± 0.051

YA	V1	PW	0.60 ± 0.0484	0.580 ± 0.049	1.092 ± 0.099	0.126 ± 0.0155	5.806 ± 1.308	1.073 ± 0.076
		RW	0.573 ± 0.0335	0.599 ± 0.039	1.135 ± 0.102	0.130 ± 0.0140	6.090 ± 1.244	1.091 ± 0.067
	V2	PW	0.589 ± 0.0552	0.581 ± 0.056	1.096 ± 0.085	0.126 ± 0.0165	5.795 ± 1.248	1.092 ± 0.071
		RW	0.561 ± 0.0479	0.593 ± 0.045	1.139 ± 0.111	0.128 ± 0.0145	6.219 ± 1.381	1.097 ± 0.058

*Note.* OA: older adult, YA: young adult, V1: visit 1, V2: visit 2, PW: pole walking, RW: regular walking. F1 impulse: foot 1 impulse, F1 peak vGRF: peak vGRF of foot 1, HS1 impulse: heel strike 1 impulse, HS1 ROL: heel strike 1 rate of loading, F1 push-off: foot 1 peak vGRF at push-off.

**Table 4:** ANCOVA results of step 1 pole force and relative time measures with step time as the covariate.

Measure	Group	Visit	Group × visit
Pole 1 impulse (N∗s)	0.55	0.46	0.26
Pole 1 peak VGRF (N)	0.66	0.72	0.57
Pole strike 1 impulse (N∗s)	0.72	0.45	0.50
Pole strike 1 ROL (N/s)	0.08	0.93	0.87
Delta1 onset (ms)	0.06	**<0.01** ^*∗*^	<0.01^*∗*^
Delta1 peak (ms)	0.27	0.02^*∗*^	0.20

*Note.* Bold *p* values represent larger mean values for visit 1 compared to visit 2 for the corresponding measure. ^*∗*^Statistical significance at *p* < 0.05.

**Table 5:** Summary of descriptive statistics (LSMeans ± SD) for all pole force and relative time measures separated by group and visit.

Group	Visit	P1 impulse (N∗s)	P1 peak vGRF (N)	PS1 impulse (N∗s)	PS1 ROL (N/s)	Delta1 onset (ms)	Delta1 peak (ms)
OA	V1	10.4 ± 6.9	31.6 ± 11.0	3.9 ± 2.7	155.6 ± 63.6	80.0 ± 55.3	20.0 ± 51.1
	V2	10.8 ± 8.3	32.4 ± 13.4	3.8 ± 2.9	159.3 ± 50.0	41.3 ± 54.5	−23.7 ± 79.9

YA	V1	13.7 ± 9.6	36.0 ± 15.5	4.3 ± 3.4	224.0 ± 142.9	32.6 ± 36.2	−24.9 ± 83.1
	V2	11.6 ± 7.1	34.0 ± 14.4	3.6 ± 2.6	222.8 ± 138.2	28.5 ± 45.3	−38.8 ± 74.4

*Note.* OA: older adult, YA: young adult, V1: visit 1, V2: visit 2, P1 impulse: pole 1 impulse, P1 peak vGRF: peak vGRF of pole 1, PS1 impulse: pole strike 1 impulse, PS1 ROL: pole strike 1 rate of loading.

**Table 6:** Partial Spearman correlation (*ρ*_partial_) coefficients with step time as the covariate.

	Delta onset		Delta peak		Pole peak vGRF		Pole strike impulse		Pole strike ROL		Pole impulse	
	*ρ*_partial_	*p* value	*ρ*_partial_	*p* value	*ρ*_partial_	*p* value	*ρ*_partial_	*p* value	*ρ*_partial_	*p* value	*ρ*_partial_	*p* value
Foot impulse	0.09	0.69	0.06	0.81	−0.25	0.27	−0.21	0.36	−0.32	0.15	−0.17	0.45
Foot peak vGRF	0.04	0.87	−0.26	0.26	−0.44	0.04^*∗*^	−0.57	0.01^*∗*^	0.06	0.78	−0.51	0.02^*∗*^
Heel strike impulse	0.28	0.22	0.00	0.99	−0.19	0.40	−0.07	0.75	−0.20	0.37	0.00	0.99
Heel strike ROL	−0.31	0.17	−0.10	0.66	−0.16	0.50	−0.27	0.23	0.01	0.95	−0.34	0.13
Foot push-off peak vGRF	0.04	0.86	−0.22	0.34	−0.41	0.07	−0.49	0.02^*∗*^	−0.13	0.57	−0.58	0.01^*∗*^

*Note.* ROL: rate of loading, vGRF: vertical ground reaction force. ^*∗*^Significant correlation between dependent measures.

## Data Availability

The data used to support the findings of this study are available from the corresponding author upon request.
